# Genomic Organization, Phylogenetic and Expression Analysis of the B-BOX Gene Family in Tomato

**DOI:** 10.3389/fpls.2016.01552

**Published:** 2016-10-19

**Authors:** Zhuannan Chu, Xin Wang, Ying Li, Huiyang Yu, Jinhua Li, Yongen Lu, Hanxia Li, Bo Ouyang

**Affiliations:** Key Laboratory of Horticultural Plant Biology (MOE), Huazhong Agricultural UniversityWuhan, China

**Keywords:** BBX, tomato, subcellular localization, phylogenetic analysis, gene expression

## Abstract

The B-BOX (BBX) proteins encode a class of zinc-finger transcription factors possessing one or two B-BOX domains and in some cases an additional CCT (CO, CO-like and TOC1) motif, which play important roles in regulating plant growth, development and stress response. Nevertheless, no systematic study of BBX genes has undertaken in tomato (*Solanum lycopersicum*). Here we present the results of a genome-wide analysis of the 29 BBX genes in this important vegetable species. Their structures, conserved domains, phylogenetic relationships, subcellular localizations, and promoter *cis*-regulatory elements were analyzed; their tissue expression profiles and expression patterns under various hormones and stress treatments were also investigated in detail. Tomato BBX genes can be divided into five subfamilies, and twelve of them were found to be segmentally duplicated. Real-time quantitative PCR analysis showed that most BBX genes exhibited different temporal and spatial expression patterns. The expression of most BBX genes can be induced by drought, polyethylene glycol-6000 or heat stress. Some BBX genes were induced strongly by phytohormones such as abscisic acid, gibberellic acid, or ethephon. The majority of tomato BBX proteins was predicted to be located in nuclei, and the transient expression assay using *Arabidopsis* mesophyll protoplasts demonstrated that all the seven BBX members tested (SlBBX5, 7, 15, 17, 20, 22, and 24) were localized in nucleus. Our analysis of tomato BBX genes on the genome scale would provide valuable information for future functional characterization of specific genes in this family.

## Background

Transcription factors are a class of proteins that regulate every aspect of plant life. They are usually composed of at least four discrete domains: DNA binding site, transcription activation domain, oligomerization site, and nuclear localization signal. All of these domains work together to control specific physiological and biochemical processes (Diao et al., 2016). Dozens of transcription factor families exist in a plant genome, such as tomato (*Solanum lycopersicum*), which represents an economically important crop and a model species for fleshy-fruit study (Tomato Genome Consortium, 2012). Among them, the B-BOX (BBX) zinc finger family drew our attention in recent years. The sequencing of plant genomes makes genome-wide analysis of the BBX gene family possible. The BBX proteins are a class of zinc-finger transcription factors possessing one or two B-BOX domains (CX_2_CX_8_CX_7_CX_2_CX_4_HX_8_H) in the N-terminal region, and the conserved Cysteine (C) and Histidine (H) residues in B-BOX domain are predicted to be involved in modulating protein-protein interactions (Khanna et al., 2009). Some BBX proteins contain an additional CCT domain near the carboxyl terminus (Khanna et al., 2009; Huang et al., 2012). The CCT domain is also highly conserved, and this domain plays important roles in transcriptional regulation and nuclear transport (Yan et al., 2011; Gendron et al., 2012). Potential segmented duplication and internal deletion events result in the differences of the consensus sequences and interspace of the zinc binding residues in the two B-BOX domains (Massiah et al., 2007; Crocco and Botto, 2013). Khanna et al. (2009) has re-identified and renamed the 32 BBX genes (*AtBBX1*~*32*) in *Arabidopsis*. These BBX genes are divided into five subfamilies based on the number of B-BOX domain and whether the protein comprises the CCT (CO, CO-like and TOC1) domain (Gangappa and Botto, 2014; Yang et al., 2014). Similar work has been conducted on rice, and 30 BBX genes (*OsBBXs*) are characterized (Huang et al., 2012).

The BBX transcription factor family is well-known to be involved in light and circadian signaling in *Arabidopsis* (Khanna et al., 2009). *CONSTANS* (*CO*) is the first identified BBX gene (known as *BBX1*) from *Arabidopsis*, which can promote flowering under long-day condition (Robson et al., 2001; Yang et al., 2014). *BBX2* and *BBX3* in *Arabidopsis* have little effect on the flowering time but over-expressing *BBX2* shortens the period of two distinct circadian rhythms (Ledger et al., 2001). Double B-BOX1a (*AtBBX18*) regulates the floral formation in *Arabidopsis* (Wang et al., 2009). Today, at least eight double B-BOX (DBB) zinc finger transcription factors have been documented to be involved in regulation of the circadian rhythm and the early photomorphogenesis in *Arabidopsis* (Kumagai et al., 2008).

BBX genes have also shown their roles in abiotic stress response. In *Arabidopsis*, the salt tolerance protein (STO, AtBBX24) has been initially identified as a protein conferring salt tolerance in yeast cells (Lippuner et al., 1996). It also can enhance *Arabidopsis* root growth under high salinity condition (Nagaoka and Takano, 2003). STO can interact with CLONE EIGHTY-ONE/RADICAL INDUCED CELL DEATH1 (CEO/RCD1) (Belles-Boix et al., 2000; Jaspers et al., 2009), which acts as a negative regulator for a wide range of stress-related genes (Fujibe et al., 2004). AtBBX18 is known as a negative regulator both in photomorphogenesis and thermotolerance (Wang et al., 2013). In *Chrysanthemum*, besides its role of delaying flowering time, *CmBBX24* can also increase plant tolerance to cold or drought (Yang et al., 2014). Among the BBX genes in rice, 29 of them possess at least one of the stress-responsive *cis*-elements (ARE, W box, GC-motif, Box-W1, HSE, and MBS), suggesting that these genes may function in response to biotic or abiotic stress (Huang et al., 2012). However, the study about BBX genes in tomato is rare.

Previously, a microarray analysis has been performed to compare the gene expression of two drought-tolerant introgression lines (IL) derived from *S. pennellii* (wild species, LA0716) and their recurrent parent *S. lycopersicum* (M82) in our laboratory (Gong et al., 2010). In the differentially-expressed gene set, a few double B-BOX (DBB) zinc finger genes have been identified, which represent a sub-class of the BBX gene family. For a better understanding of the BBX genes in tomato, we investigated all the 29 BBX members of tomato, including their gene structures, phylogenetic relationships, subcellular localizations, tissue expression profiles, and expression patterns under various hormones and stress treatments. Our analysis of tomato BBX genes at the genomic level would provide a solid basis for further functional characterization of specific genes in the family.

## Materials and methods

### Sequence retrieval

As *Arabidopsis* BBX family has been reported previously, all the protein sequences from the family were extracted from the *Arabidopsis* Information Resource (TAIR) database (http://www.arabidopsis.org) and used as queries for BLASTP search with an e-value threshhold of <1e-5 in the NCBI database (http://www.ncbi.nlm.nih.gov/pubmed) to identify the homologous in tomato. Afterward, they were confirmed using BLASTN search against the Sol Genomics Network database (SGN; https://www.sgn.cornell.edu). Sequences of BBXs from other species were also retrieved from the NCBI database.

### Phylogenetic analysis and sequence alignment

Multiple sequence alignments of BBX proteins were generated using ClustalW in BioEdit (version 7.2.5, http://bioedit.software.informer.com), and the phylogenetic tree was constructed with the neighbor-joining algorithm in MEGA (version 5.1) (Tamura et al., 2011). Bootstrap analysis was carried out with 1000 replicates. Domains were identified with SMART (http://smart.embl-heidelberg.de), pfam (http://pfam.xfam.org) and InterProscan (http://www.ebi.ac.uk/interpro/search/sequence-search) programs. WebLogo (http://weblogo.berkeley.edu/logo.cgi) was used to generate the sequence logos of conserved domains (Crooks et al., 2004).

### Chromosomal location, gene structure, and duplication analysis

BBX genes were mapped to tomato chromosomes by identifying their chromosomal positions according to the SGN database (Tomato Genome Consortium, 2012). Accordingly, their cDNA sequences and the corresponding genomic DNA sequences of BBX members were obtained, then exons and introns were identified by comparing the genomic DNA and cDNA sequences using Gene Structure Display Server (GSDS; http://gsds.cbi.pku.edu.cn) (Guo et al., 2007). Duplication analysis was also conducted on tomato BBX gene family.

### *Cis*-element prediction for BBX gene promoter

The sequences of B-BOX were used as queries in BLASTN searches against the tomato whole genome scaffolds data (version 2.40) at the SGN website. The promoter sequences (2 kb upstream of 5′UTR) of all the annotated BBX genes were submitted to the PlantCARE database (http://bioinformatics.psb.ugent.be/webtools/plantcare/html/) for *cis*-element prediction.

### Plant growth condition, hormone and stress treatment

Tomato seeds (*S. lycopersicum* cv. Alisa Craig) were geminated at 28°C in petri dishes containing moist filter papers. Three days later, uniform germinated seeds were transferred to a self-made germination device, which is constituted of a layer of sponge and a tray with 15 liters of modified Hoagland's nutrient solution (see in Table S3) (Urbanczyk-Wochniak and Fernie, 2005). After 1 week, seedlings were transplanted to plastic trays with 30 liters of modified Hoagland's solution, which was well aerated at 1 h interval with an air pump. The seedlings were grown at 25 ± 2°C in a growth room under a photosynthetic photon flux density of approximate 3500 Lux and with a 16 h/8 h light/dark cycle.

One-month-old seedlings were used to investigate the effects of abiotic stresses and hormone treatments on BBX gene expression (Zhu et al., 2012; Gupta et al., 2013). Seedlings were treated with hydroponic solution (pH 6.2) containing 5 μM Abscisic acid (ABA), Gibberellic acid (GA_3_), Auxin (IAA), 6-Benzylaminopurine (6-BA), Methyl jasmonate (MeJ), Brassinolide (BR), Salicylic acid (SA), Methylviologen (MV) or 50 μM ethephon (ETH), 10% polyethylene glycol-6000 (PEG-6000), and 100 mM NaCl. Control plants were mock-treated with hydroponic solution only. Shoots (including stem and leaf tissue) were collected from both treated and control plants at 0, 0.25, 0.5, 1, 2, 6, 12, and 24 h. Heat and cold stresses were applied by placing seedlings in 42 and 4°C growth chamber, respectively. Shoots were harvested at 0, 3, 6, 12, and 24 h post heat stress, and 0, 1.5, 6, 12, and 24 h post cold stress. Drought stress was performed by pulling out the whole plants and placing them on a clean bench, and temperature and humidity were well-controlled by a central air-conditioning system (Lu et al., 2012). Then, the shoots were collected 0, 0.5, 1, 1.5, 2, and 2.5 h later. The untreated plants at the same time points were used as the corresponding controls in order to avoid the effects of circadian clock on gene expression difference.

To investigate gene expression profiles at the organ-specific level, samples including roots, stems, leaves, flowers, and fruits at different stages were collected from 5-month-old tomato plants.

Aiming at exploring the difference of BBX gene expression between *S. pennellii* (LA0716) and cultivated tomato (M82) under drought stress, drought treatment as described above was also applied on these two tomato species, while the shoots were collected only at 1 and 2.5 h post the treatment.

All samples were collected in triplicate from each of the sampling points. The samples were frozen in liquid nitrogen and stored at −80°C until use.

### Real-time qRT-PCR analysis

Total RNA from all the samples described above was extracted with TriZol reagent (Invitrogen, Gaithersburg, MD, USA) according to the manufacturer's instruction. To remove residual genomic DNA, the RNA samples were treated with RNase-free DNase I (Invitrogen). Approximate four micrograms of DNase-treated total RNA were used for the first-strand cDNA synthesis with M-MLV reverse transcriptase (Invitrogen) and oligo dT (Promega, Madison, WI, USA). The reverse-transcribed product (40 μl) was diluted to a final volume of 500 μl and used for quantitative PCR. Primers were designed from tomato BBX sequences using primer 3.0 online (All primers are listed in Table S1). The specificity of each pair of primers was checked by dissociation curve analysis. Real-time PCR was performed in an optical 96-well plate with a LightCycler 480 instrument (Roche diagnostics, Basel, Switzerland). Each reaction contained 5 μl SYBR premix Ex Taq (Takara, Kyoto, Japan), 2 μl cDNA samples, and 0.5 μl of each primer (10 μM) in a reaction system of 10 μl. The thermal cycle was as follows: 95°C for 1 min, 40 cycles of 95°C for 10 s, 58°C for 15 s, and 72°C for 20 s. Three technical replicates were performed for each sample. Three reference genes: Elongation factor 1α (*EF1*), protein phosphatase 2A (*PP2Acs*), and actin (*ACT*) were used (Fuentes et al., 2016), and finally the tomato actin gene was used in the calculation of relative expression level due to its wide application in tomato expression profiling and superior performance in our study. Real-time PCR data were analyzed using the 2^ΔΔCt^ method (Schmittgen and Livak, 2008). Gene expression data were log2 transformed before analysis, and heatmaps were generated by R software package “pheatmap” (Ihaka and Gentleman, 1996).

### Subcellular localization analysis

The subcellular location of tomato BBX proteins was predicted using WoLF PSORT (http://www.genscript.com/psort/wolf_psort.html). To evaluate the prediction results, seven BBX genes were selected and their coding sequences without termination codon were amplified from the cDNAs of Alisa Craig. The amplified products were cloned into pENTR™/SD/D-TOPO entry vector (Invitrogen), and then transferred to the plant Gateway vector pGWB451 by LR recombination reaction. This binary vector enables the transient expression of a target protein *via* C-terminal fusion to the green fluorescent protein (GFP) (Nakagawa et al., 2009). Meanwhile, the cyan fluorescent protein (CFP) labeled *Ghd7* was used as a nuclear marker in the co-localization analysis (Xue et al., 2008). *Arabidopsis* protoplasts from young leaves were prepared and transformed with the vectors described above according to a previous study (Yoo et al., 2007). Fluorescence images were captured and analyzed using a Zeiss laser scanning confocal microscope LSM 510 META (Carl Zeiss, Jena, Germany) and the LSM image software.

## Results

### Identification of BBX genes in tomato

To obtain a global view of the BBX genes in tomato genome, keyword and BLAST searches were performed at SGN, NCBI, and other public databases. After removing the redundant sequences, 29 putative BBX genes were identified. For the sake of consistency, we named these genes as *SlBBX1* to *SlBBX29* according to their homology to the BBX members of *Arabidopsis* firstly, and then the rest members were named depending on their homology to the newly named tomato BBX genes. The detailed information of each BBX was presented in Table 1, including gene name, Soly ID, chromosome location, genomic position, length of coding sequence and protein, theoretical isoelectric point, and molecular weight. The 29 deduced proteins had divergent lengths, resulting in diverse isoelectric points and molecular weights. The length of the coding sequences ranged from 267 (*SlBBX18*) to 1428 bp (*SlBBX27*). The tomato BBX genes encode proteins with predicted molecular weights of 9.6–53.1 kDa and theoretical isoelectric points from 4.49 (*SlBBX7*) to 9.39 (*SlBBX26*). The majority of tomato BBX proteins was predicted to be located in nuclei, but a few of them may be located in other subcellular compartments, such as chloroplast and cytoplasm.

**Table 1:** **The detailed information of SlBBX members**.

**Gene**	**Annotated CDS**	**Genomic position**	**Chr**	**CDS**	**AA**	**pIs**	**MW**	**Subcellular localization**
SlBBX1	Solyc02g089520.1	45892058-45893830	2	1230	409	5.07	45.67	nucl: 12, chlo: 1
SlBBX2	Solyc02g089500.2	45885737-45886444	2	429	142	8.50	15.18	nucl: 6, mito: 5, chlo: 1, cyto: 1
SlBBX3	Solyc02g089540.2	45900821-45903523	2	1176	391	5.57	43.43	nucl: 10, chlo: 2, cyto: 2
SlBBX4	Solyc08g006530.2	1142836-1144790	8	1050	349	5.28	38.65	nucl: 8, chlo: 3, cyto: 3
SlBBX5	Solyc12g096500.1	63736405-63737572	12	1077	358	5.53	39.50	chlo: 10, nucl:3
SlBBX6	Solyc07g006630.2	1494827-1496702	7	1161	386	6.36	42.60	chlo: 7, cyto: 4, nucl: 1, mito: 1
SlBBX7	Solyc12g006240.1	756961-761422	12	810	269	4.49	29.70	nucl: 9, chlo: 3, mito: 1
SlBBX8	Solyc05g020020.2	25874229-25879668	5	1233	410	5.50	44.52	nucl: 11, cyto: 2
SlBBX9	Solyc07g045180.2	55614263-55620975	7	1257	418	5.41	46.13	nucl: 14
SlBBX10	Solyc05g046040.1	57316966-57319191	5	1260	419	4.97	46.26	nucl: 11, mito: 1, E.R.: 1
SlBBX11	Solyc09g074560.2	61872048-61874336	9	1122	373	5.80	42.20	nucl: 9, cyto: 3, chlo: 1
SlBBX12	Solyc05g024010.2	30237066-30240376	5	1359	452	6.79	49.70	nucl: 11, cyto: 1, extr: 1
SlBBX13	Solyc04g007210.2	904265-906430	4	1287	428	5.33	48.71	nucl: 11, chlo: 1, mito: 1
SlBBX14	Solyc03g119540.2	62171978-62173730	3	1227	408	5.17	46.69	nucl: 11, chlo: 1, mito: 1
SlBBX15	Solyc05g009310.2	3442971-3445112	5	1314	437	5.53	49.81	nucl: 7, chlo: 3, mito: 2, cyto: 1
SlBBX16	Solyc12g005750.1	400112-400441	12	330	110	7.99	12.88	cyto: 8, nucl: 3, chlo: 1, mito: 1
SlBBX17	Solyc07g052620.1	58408926-58409318	7	393	130	8.58	14.51	nucl: 8, chlo: 3, cyto: 2
SlBBX18	Solyc02g084420.2	42107507-42109459	2	267	88	5.47	9.57	cyto: 12, chlo: 1
SlBBX19	Solyc01g110370.2	88848214-88852895	1	726	241	5.31	26.87	cyto: 6.5, cyto_nucl: 5.5, nucl: 3.5, chlo: 1, plas: 1, cysk: 1
SlBBX20	Solyc12g089240.1	62792418-62794690	12	990	329	7.04	36.39	nucl: 10, chlo: 2, chlo: 1
SlBBX21	Solyc04g081020.2	62677396-62678694	4	900	299	7.51	33.25	nucl: 7, cyto: 4, extr: 2
SlBBX22	Solyc07g062160.2	62191788-62196800	7	897	311	4.89	33.66	nucl: 10, chlo: 3
SlBBX23	Solyc12g005420.1	251564-253391	12	849	282	5.87	30.63	chlo: 9, nucl: 4
SlBBX24	Solyc06g073180.2	41479688-41483031	6	702	233	4.97	25.91	nucl: 6, cyto: 4, chlo: 1, plas: 1, extr: 1
SlBBX25	Solyc01g110180.2	88706256-88707726	1	612	203	5.63	22.62	nucl: 9, cyto: 2, extr: 2
SlBBX26	Solyc10g006750.2	1205986-1206786	10	315	104	9.39	12.03	nucl: 10.5, cyto_nucl: 6.5, cyto: 1.5
SlBBX27	Solyc04g007470.2	1146534-1149345	4	1428	475	5.66	53.13	nucl: 14
SlBBX28	Solyc12g005660.1	351057-351746	12	609	202	4.89	22.26	chlo: 8, nucl: 2, cyto: 2, extr: 2
SlBBX29	Solyc02g079430.2	38574418-38575502	2	558	185	4.72	20.72	nucl: 9, chlo: 2, cyto: 1, plas: 1

*Chr, chromosome; CDS, length of coding sequence; AA, number of amino acid; pIs, theoretical isoelectric point; MW, molecular weight, KDa; The subcellular location of tomato BBX proteins was predicted using WoLF PSORT (http://www.genscript.com/psort/wolf_psort.html). Nucl, nucleus; Mito, mitochondria; Chlo, chloroplast; Cyto, cytosol; E.R, endoplasmic reticulum; Cysk, cytoskeleton; Plas, plasma membrane; Extr, extracellular. Testk used for kNN is: 14*.

### Protein sequence, phylogenetic, and duplication analysis of the tomato BBX family

**Figure 1:**
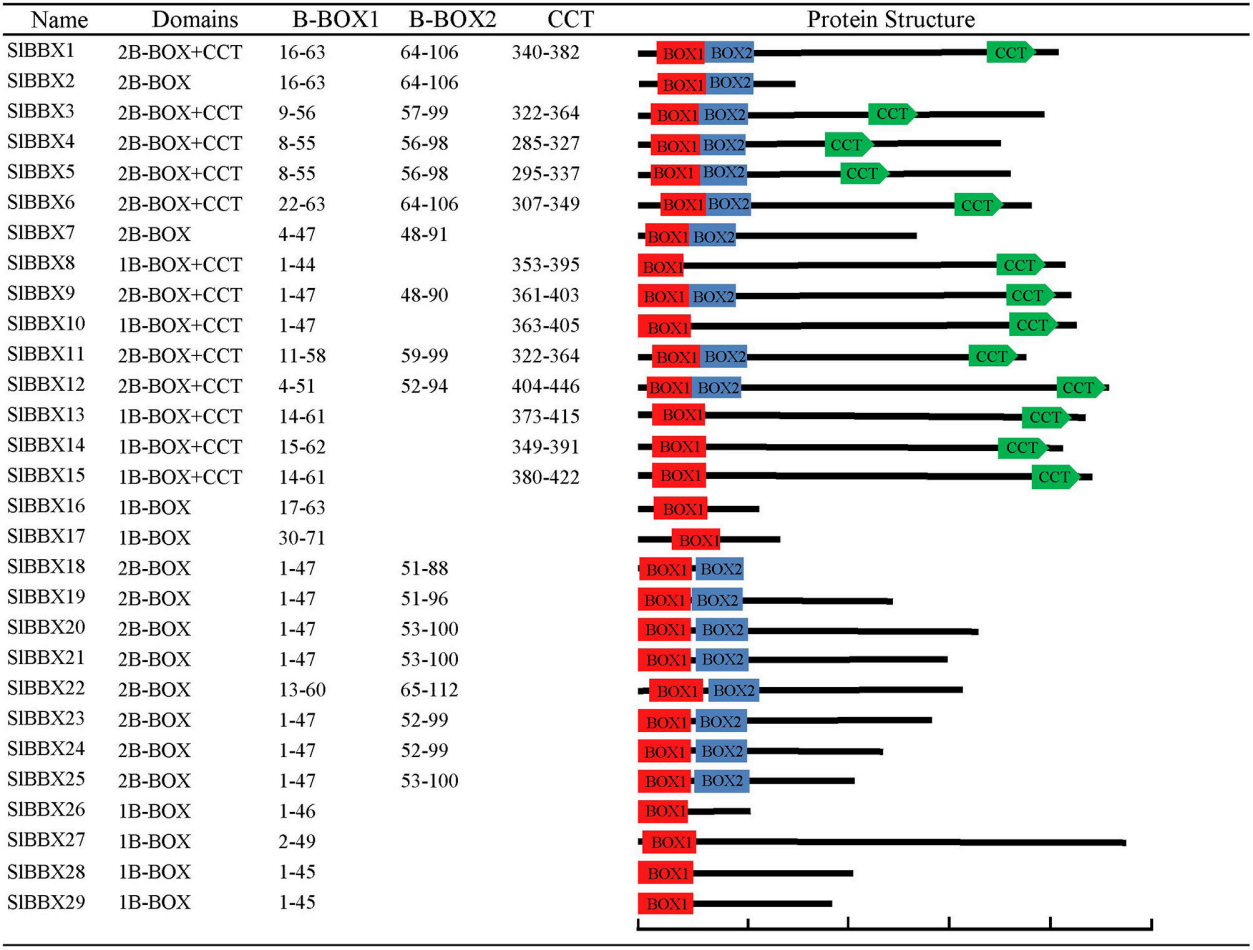
**Structure of the tomato BBX proteins**. Numbers indicate amino acid position of the corresponding conserved domains. The red, blue rectangles and green pentagons indicate the B-BOX1, B-BOX2 and CCT domain, respectively. The scale bar represents 100 amino acids.

The tomato BBX proteins varied widely from 88 to 475 amino acids. Among them, eight SlBBXs contained two B-BOX domains and a conserved CCT domain. Ten members contained two B-BOX domains but no CCT domain. Six SlBBXs consisted of only one B-BOX domain, and five with one B-BOX domain plus a CCT domain (Figure 1). The sequences of the conserved domain B-BOX1, B-BOX2, and CCT are shown in Figure S2. It is clear that some amino acid residues were more conserved than others in those domains, for example, the cysteine residues constituting the zinc finger were highly conserved in the B-BOX domains. The protein sequence alignment results indicated that the two B-BOX domains had similar conserved sequences, and the CCT domain across the whole family was highly conserved.

**Figure 2:**
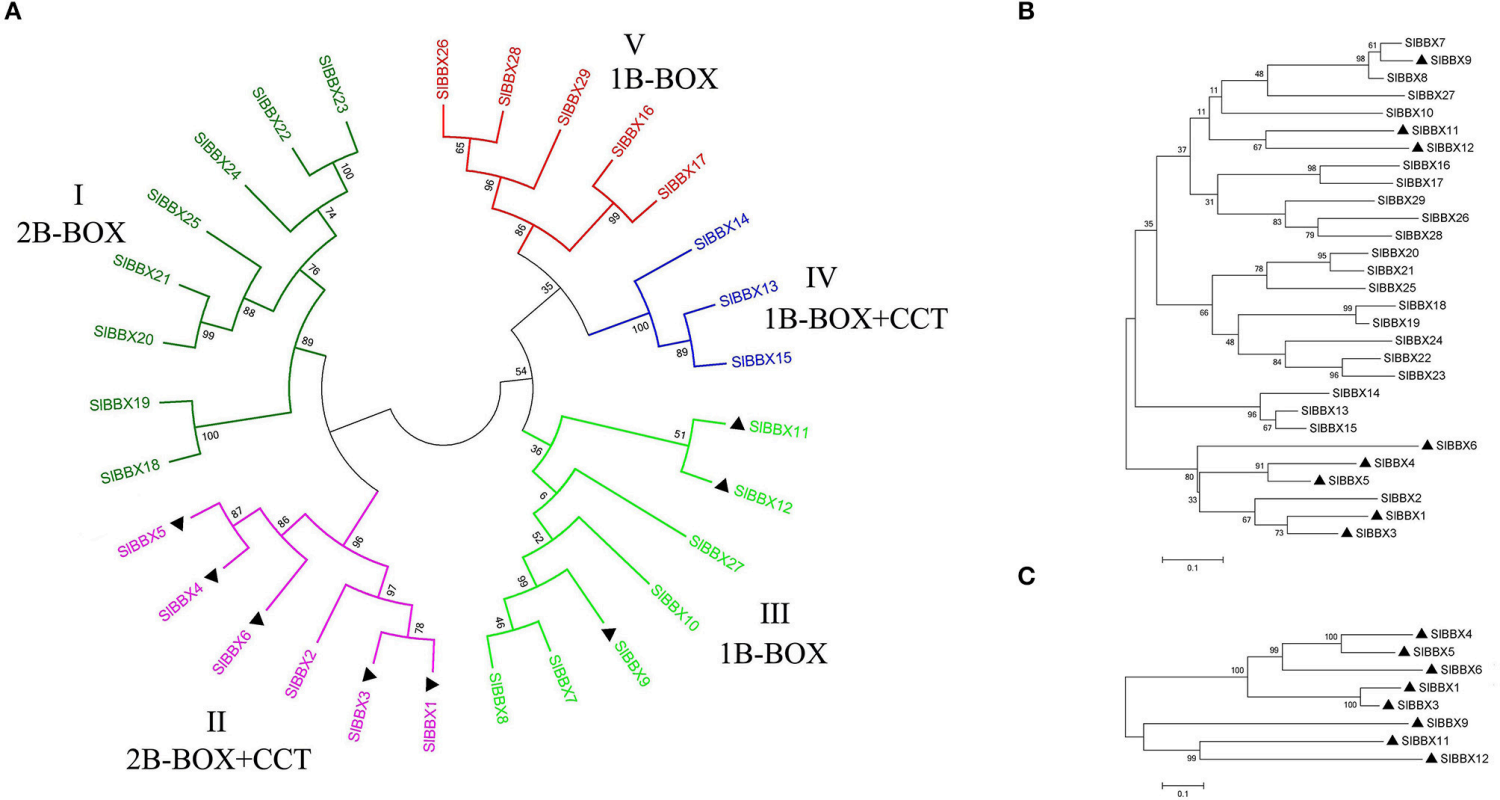
**Phylogenetic analysis of the tomato BBX family**. The trees shown are based on the alignments of the protein sequences of the full length **(A)**, the B-BOX 1 domain **(B)**, and two B-BOX plus the CCT domains **(C)**. The bootstrap values are indicated at each node. The scale bar represents 0.1 amino acid substitutions per site. The members marked in black triangle contain two B-BOX and one CCT domains.

To explore the phylogenetic relationship and divergence of the BBX family in tomato, a phylogenetic tree was constructed with MEGA5 according to the aligned 29 BBX protein sequences (Figure 2). The phylogenetic tree of SlBBXs, together with AtBBXs and OsBBXs was also constructed (Figure S1). As shown in the phylogenetic tree, most BBX members from *Arabidopsis* and tomato clustered together. In order to know further the phylogenetic relationship, alignments were also performed for the sequences of the first B-BOX domain (Figure 2B), and the two concatenated B-BOX domains plus the CCT domain (Figure 2C), respectively. The tomato BBX family was divided into five subfamilies based on the phylogenetic analysis and previous studies in *Arabidopsis* and rice (Khanna et al., 2009; Huang et al., 2012). Among them, the members from subfamily I contained two concatenated B-BOX domains, while the members from subfamily II owned two B-BOX domains plus a CCT domain except for SlBBX2, which only contained two B-BOX domains. In the subfamily III, *SlBBX8* and *SlBBX10* contained one B-BOX domain plus the CCT domain, and the other members contained two B-BOX domains with (*SlBBX9, SlBBX11*, and *SlBBX12*) or without (*SlBBX7*) the CCT domain. All the members in subfamily IV possessed one B-BOX domain and a CCT domain, and the members of subfamily V just contained one B-BOX domain. Basically, the phylogenetic tree constructed from the 29 SlBBXs (Figure 2A) was similar to that constructed based on the first B-BOX domain (Figure 2B). Eight SlBBXs from subfamily II and III contained all the three domains, and they could be divided into two groups (Figure 2C). Five of them (*SlBBX1, 3, 4, 5, 6*) from subfamily II aligned together, while the other three members (*SlBBX9, 11, 12*) from subfamily III were also clustered together.

### Chromosomal localization, gene structure, and duplication

**Figure 3:**
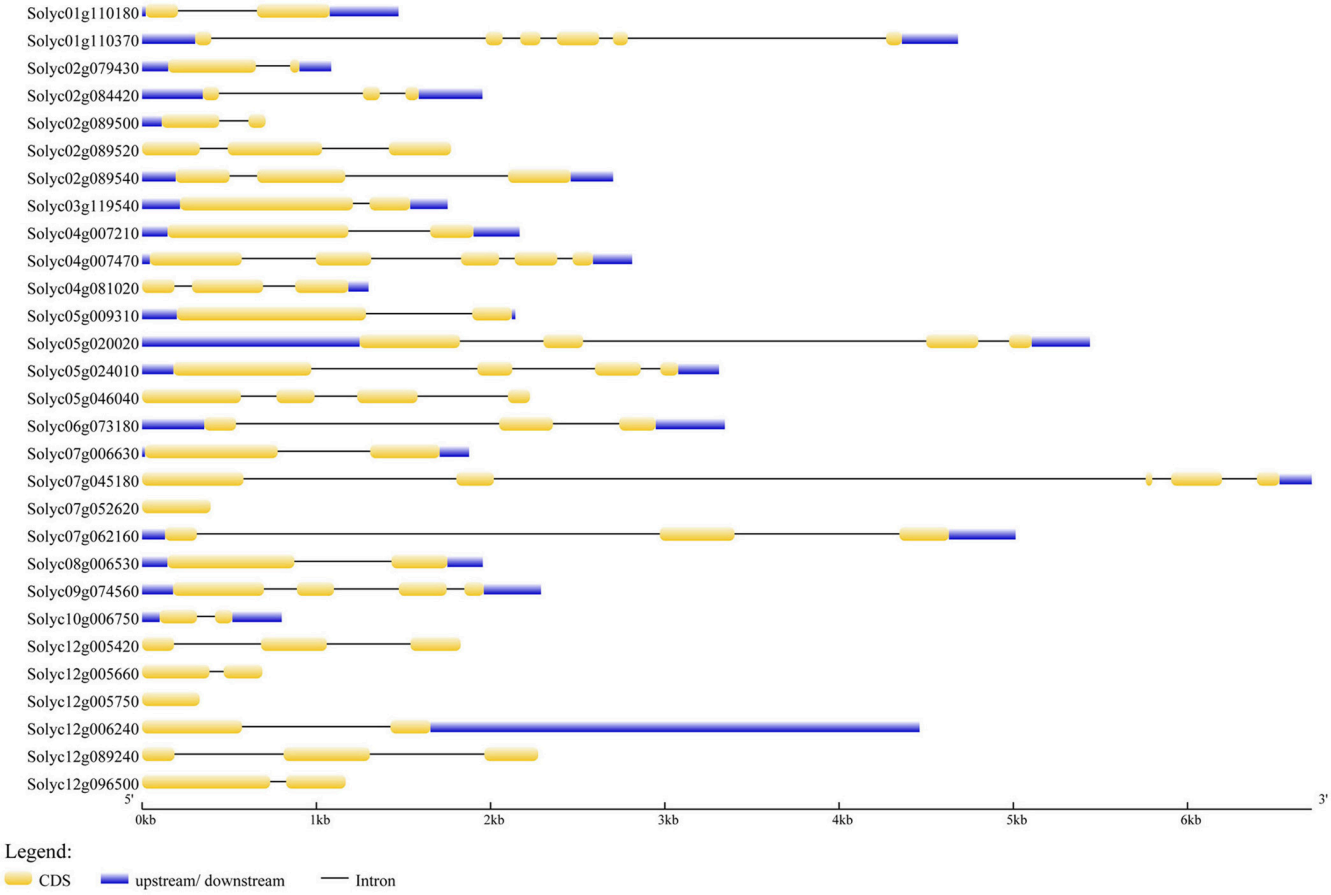
**Gene structure of the tomato BBX family generated from GSDS**. The yellow block means the coding sequence (CDS), the blue block means the upstream or downstream of the genes, and the black line indicates the intron. The scale bar indicates the length of the DNA sequences.

**Figure 4:**
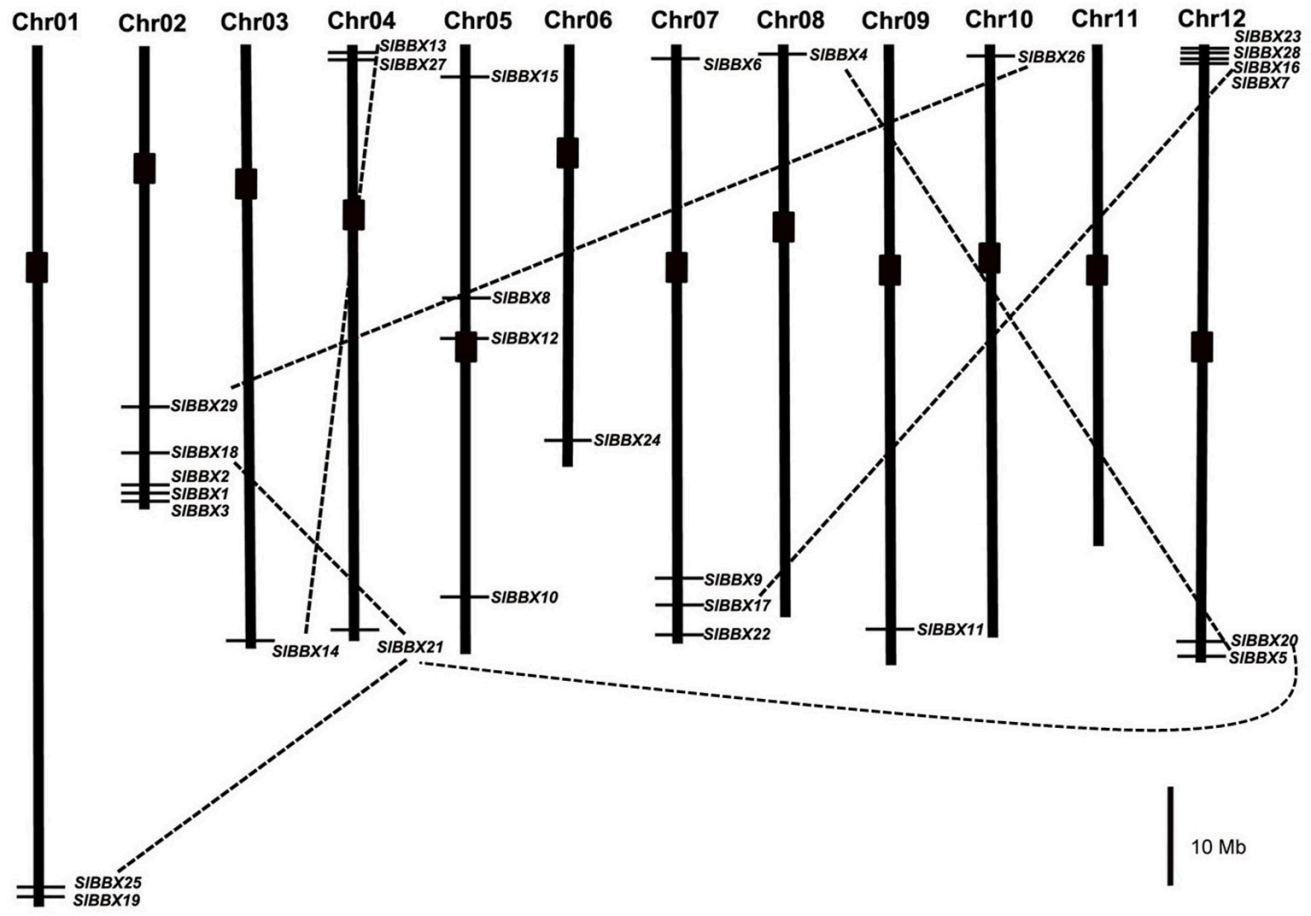
**Chromosome distribution and duplication events of tomato ***BBX*** genes**. Chromosomal mapping was based on the physical position (Mb) in 12 tomato chromosomes. The chromosome number is indicated at the top of each bar. The positions of the tomato BBX genes in the chromosomes were obtained from Sol Genomics Network database (SGN, https://www.sgn.cornell.edu). 7 pairs of paralogous gene connected with black dotted lines represent segmental duplication. The black blocks indicate the positions of the centromeres in chromosomes, respectively. Scale represents 10 Mb chromosomal distance.

To determine the genomic distribution of tomato BBX genes, they were mapped to chromosomes of the published tomato genome (**Figure 4**). BBX genes were distributed on all chromosomes, except for chromosome 11. Among them, only one BBX gene was distributed on chromosome 3, 6, 8, 9, and 10; two on chromosome 1, three on chromosome 4, four on chromosome 5 and 7, five on chromosome 2, and six on chromosome 12. On chromosome 2, three BBX genes (*SlBBX1, 2*, and *3*) were found located within the chromosome region corresponding to the introgressed segment of the drought-tolerant introgression line, IL2-5. In addition, the gene structures of BBX members were plotted with the GSDS software (Figure 3). Most BBX genes had one to five introns, except for *SlBBX16* and *SlBBX17* which had no intron. Among the tomato BBX genes, 12 were found to be segmentally duplicated, but none of them are arranged in tandem (Figure 4).

### *Cis*-elements in the promoters of tomato BBX genes

As *cis*-elements are involved in gene regulation by interacting with their corresponding trans-regulatory factors, therefore, studies on the putative *cis*-elements would provide valuable information for the expression of tomato BBX genes. So the promoter regions of all the BBX genes were retrieved and submitted to PlantCARE database for *cis*-elements identification (Table 2, Table S2). A total of 26 *cis*-elements were identified. As expected, the conventional promoter elements (TATA-box, CAAT-box) were detected in all the SlBBXs promoters. The remaining 24 *cis*-acting elements can be divided into four groups. Fourteen *cis*-elements are light responsive, including Box I, G-Box, Box 4, I-box, GT1-motif, circadian, AE-box, GATA-motif, SP1, ACE, TCT-motif, GA-motif, GAG-motif and ATCT-motif. Five *cis*-elements are hormone responsive, including ERE, ABRE, CGTCA-motif, TGACG-motif, and TCA-element. Four of them are well-known as stress responsive elements: HSE, TC-rich repeats, MBS, and ARE. The fourth group has only one *cis*-element (Skn-1_motif) which is required for endosperm expression, this element was identified in the promoters of 26 BBX genes.

**Table 2:** **The ***cis***-elements identified in the promoters of more than ten SlBBX genes**.

***Cis*-elements**	**Number of genes**	**Functions of *cis*-elements**	**Type of *cis*-elements**
CAAT-box	29	Common *cis*-acting element in promoter and enhancer regions	
TATA-box	29	Core promoter element around –30 of transcription start	
Skn-1_motif	26	*Cis*-acting regulatory element required for endosperm expression	Endosperm expression
Box I	24	Light responsive element	Light responsive
HSE	22	*Cis*-acting element involved in heat stress responsiveness	Stress responsive
TC-rich repeats	22	*Cis*-acting element involved in defense and stress responsiveness	Stress responsive
Box 4	22	part of a conserved DNA module involved in light responsiveness	Light responsive
G-Box	21	*Cis*-acting regulatory element involved in light responsiveness	Light responsive
ERE	20	Ethylene-responsive element	Hormone responsive
ABRE	19	*Cis*-acting element involved in the abscisic acid responsiveness	Hormone responsive
GT1-motif	19	Light responsive element	Light responsive
I-box	18	Part of a light responsive element	Light responsive
MBS	17	MYB binding site involved in drought-inducibility	Stress responsive
circadian	17	*Cis*-acting regulatory element involved in circadian control	Light responsive
GATA-motif	17	Part of a light responsive element	Light responsive
AE-box	16	Part of a module for light response	Light responsive
Spl	16	Light responsive element	Light responsive
ACE	16	*Cis*-acting element involved in light responsiveness	Light responsive
CGTCA-motif	15	*Cis*-acting regulatory element involved in the MeJA-responsiveness	Hormone responsive
TCA-element	15	*Cis*-acting element involved in salicylic acid responsiveness	Hormone responsive
TGACG-motif	14	*Cis*-acting regulatory element involved in the MeJA-responsiveness	Hormone responsive
TCT-motif	14	Part of a light responsive element	Light responsive
GA-motif	14	Part of a light responsive element	Light responsive
ARE	12	*Cis*-acting regulatory element essential for the anaerobic induction	Stress responsive
GAG-motif	11	Part of a light responsive element	Light responsive
ATCT-motif	11	Part of a conserved DNA module involved in light responsiveness	Light responsive

### The differences of *cis*-elements in promoters and expression patterns of BBXs under drought stress between *S. pennellii* and M82

*S. pennellii* LA0716 is tolerant to drought and salt stress, while the cultivated tomato M82 is sensitive to stress (Bolger et al., 2014). And a previous study indicates possible involvement of BBX genes in drought response of tomato (Gong et al., 2010). In order to know the expression differences of BBX genes between the wild species *S. pennellii* (LA0716) and cultivated tomato (M82), *cis*-elements in the promoter region of each BBX gene were analyzed, and the expression patterns of all the BBX genes under drought treatment were also investigated (Table S4, Figure S3). It was shown that every BBX gene of *S. pennellii* and M82 carried different *cis*-elements in their promoter regions (Table S4). For instance, ABRE and TCA elements were identified in *SpBBX1* promoter but they were lacking in the promoter of *SlBBX1* in M82, and these elements are well-known to be involved in ABA and SA response respectively. The major different *cis*-elements of the whole family were related to light, hormone, and stress response.

We further compared the expression of BBX genes between LA0716 and M82 under drought stress. According to the real-time PCR results, the 29 *SlBBXs* could be divided into five major clusters (Figure S3). *SlBBX15* stood alone as a class, it showed a similar and unchanged level in the first hour of drought stress between LA0716 and M82, and however, upon 2.5 h of treatment, it was decreased in M82 while dramatically increased in LA0716. The expression of the genes in class II showed a similar increasing pattern upon drought stress in both genotypes, while some of the genes (*SlBBX16, 22*, and *25*) response more quickly in M82. The expression of genes in class III was relatively higher in LA0716 at 2.5 h than that in M82. The transcripts of the genes in class IV kept increasing in M82 but they were remained largely unchanged in LA0716. The expression patterns of the genes in class V were opposite, which was induced in M82 but down-regulated in LA0716.

### Organ-specific expression of BBX genes in tomato

**Figure 5:**
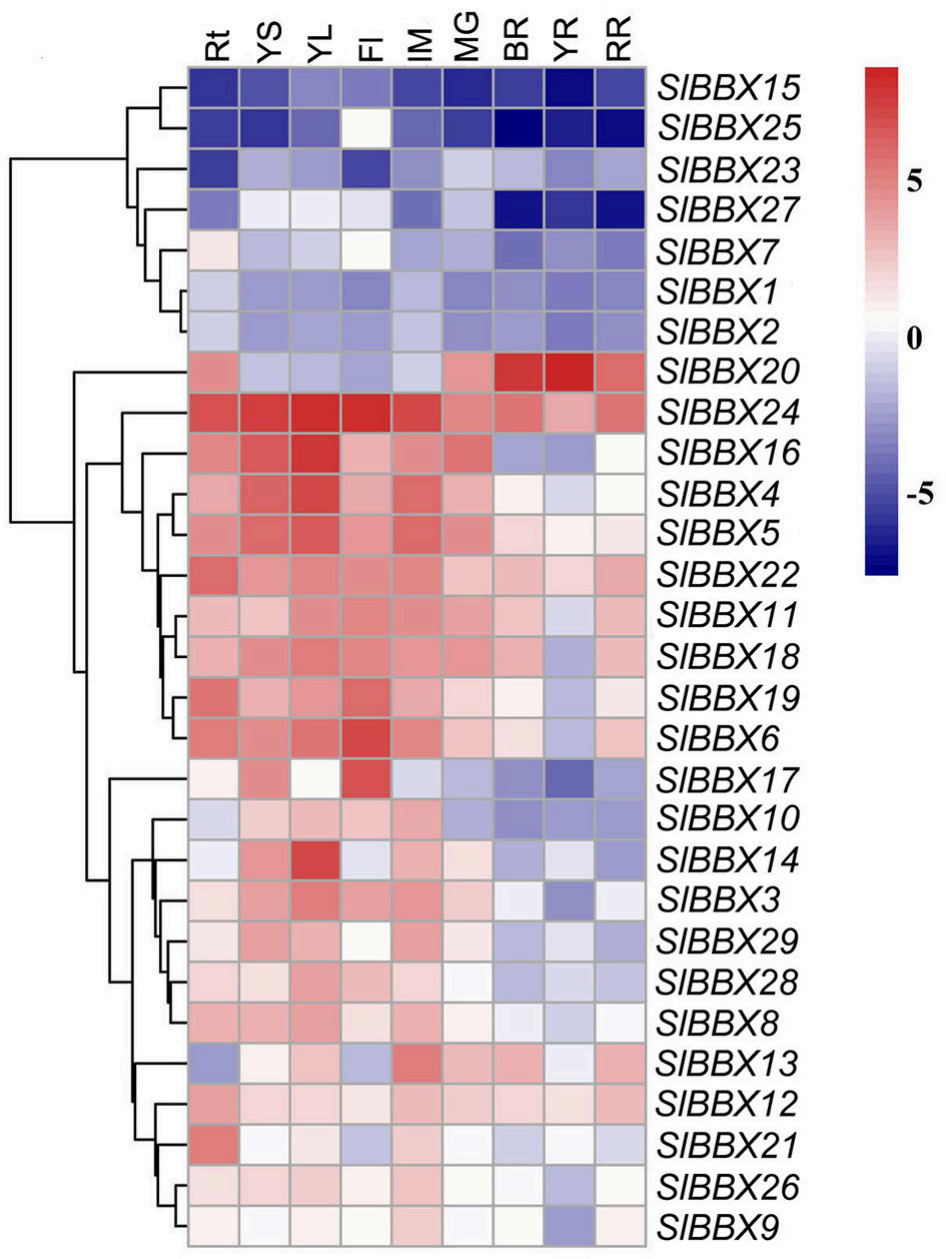
**Expression profiles of tomato BBX genes in various organs**. The relative expression data were log2 transformed with R software, and a cluster dendrogram is shown on the left of the heat map. Blocks with blue colors indicate decreased and red ones indicate increased transcription levels. Rt, root; YS, young stem; YL, young leaf; Fl, flower; IM, immature fruit; MG, mature green fruit; BR, breaker fruit; YR, yellow ripe fruit; RR, red ripe fruit.

To investigate the tissue expression pattern of all the BBX genes, qRT-PCR was carried out on the cultivated tomato Alisa Craig, with actin gene as the endogenous control. As shown in Figure 5, different members of the tomato BBX family showed distinct expression patterns. *SlBBX24* was constitutively expressed at a high level in nearly all tissues tested, and seven BBX genes (*SlBBX1, 2, 7, 15, 23, 25*, and *27)* were also expressed constitutively but with low transcript abundance. *SlBBX20* was preferentially expressed in fruit and root tissue, and eight genes (*SlBBX4, 5, 6, 11, 16, 18, 19*, and *22*) showed relatively higher expression levels in vegetative tissues, immature fruit, and mature green fruit, but with low expression at ripening stage. A similar expression pattern was observed on six BBX genes (*SlBBX3, 8, 10, 14, 28*, and *29*), which showed relatively higher expression levels in young stem, young leaf and immature fruit, but a lower level of expression in mature fruit. With a slight difference to the above six genes, *SlBBX17* had a higher expression in young stem and flower, and a low level expression in fruit. *SlBBX13* was expressed at a low level in root, flower and yellow fruit. The other BBX genes were specifically expressed in one or several organs, such as *SlBBX9, 12, 21*, and *26*.

### Expression of BBX genes in response to exogenous hormones

**Figure 6:**
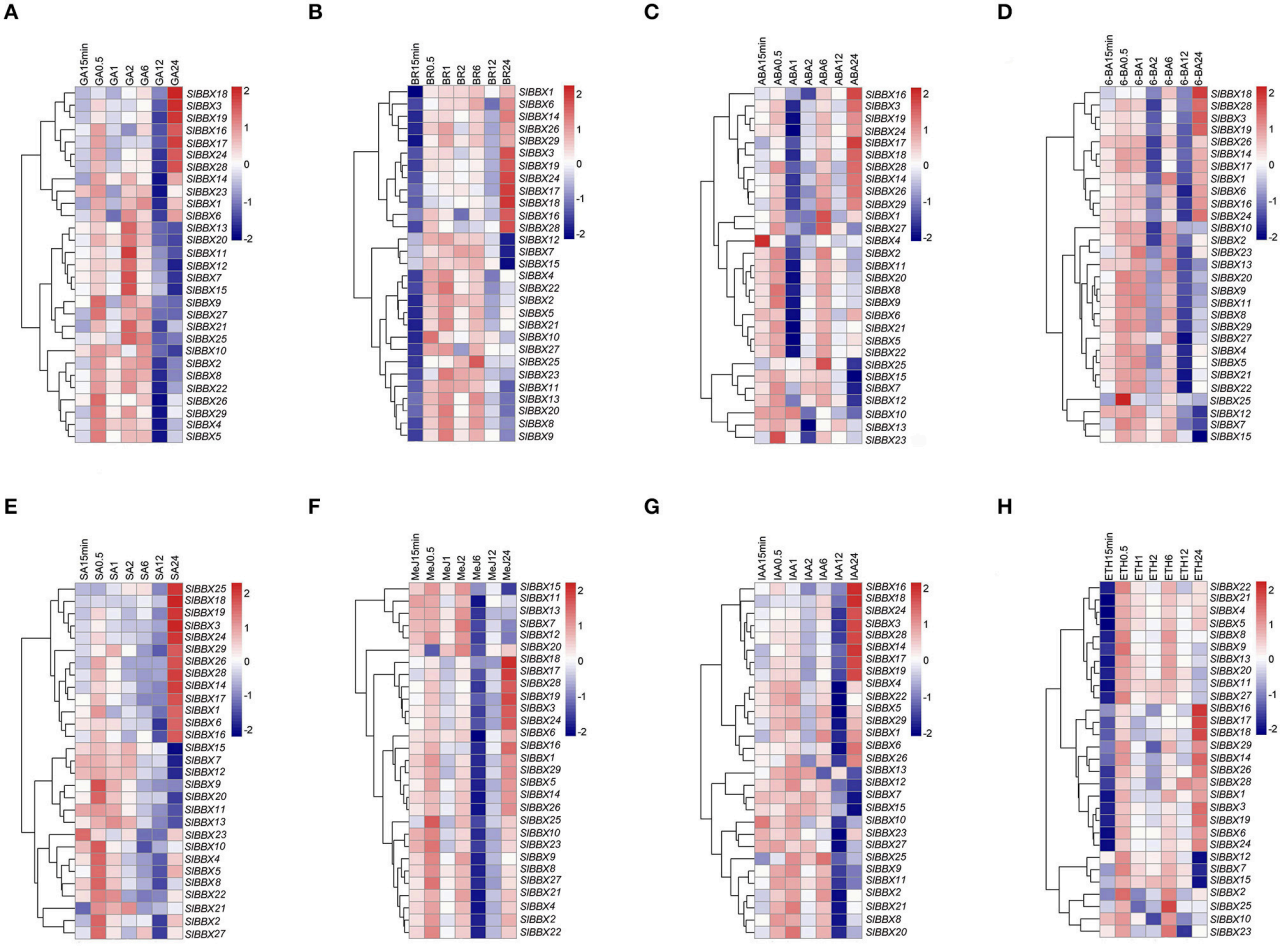
**Expression profiles of tomato BBX genes under treatments of exogenous hormones in hydroponic culture**. The relative expression data were log2 transformed with R software, and a cluster dendrogram is shown on the left of each heat map. Blocks with colors indicate decreased (blue) or increased (red) transcript level relative to the corresponding control (Plants grown in hydroponic solution at the same time without hormone treatments). **(A–H)** represent the gene expression patterns of BBX genes detected using real-time PCR under eight different hormone treatments. CK, GA, BR, ETH, ABA, 6-BA, SA, MeJ, and IAA represent seedlings treated with water, 5 μM gibberellic acid, 5 μM brassinosteroids, 50 μM ethephon, 5 μM abscisic acid, 5 μM 6-benzylaminopurine, 5 μM salicylic acid, 5 μM methyl jasmonate, and 5 μM auxin, respectively. The numbers 0.5, 1, 2, 6, 12, and 24 indicate the time (hour) after treatments, and 15 min represents 15 min after treatment.

Hormones play vital roles in plant growth and development. To examine the effects of various hormones on the expression of tomato BBX gene family, qRT-PCR was used to analyze the transcriptional levels of all the BBX genes under eight hormone treatments (Figure 6). Under GA treatment (Figure 6A), most BBX genes showed an increased expression level at 0.5 and 2 h after GA application. The transcripts of all the genes decreased at 12 h, and more than half of them maintained at a low level at 24 h. However, seven genes (*SlBBX3, 16, 17, 18, 19, 24*, and *28*) showed a drastic increase from 12 to 24 h. Six *SlBBXs* (*SlBBX7, 11, 12, 13, 15*, and *20*) showed a similar expression pattern, which was induced by exogenous GA and had a peak at 2 h after GA treatment, then the transcription abundance of them were down-regulated with time. As for BR treatment (Figure 6B), the expression of most BBX genes was inhibited at the beginning (15 min) and then induced by BR during the following several hours. A large proportion of the genes were down-regulated at 12 h post BR application, while some of them were up-regulated later (*SlBBX3, 16, 17, 18, 19, 24*, and *28*), and some were further down-regulated (such as *SlBBX7, 12*, and *15*). When the plants were challenged with ABA (Figure 6C), most BBX genes were induced at the very beginning (especially *SlBBX4*), but they were down-regulated at 1 h. After that, the transcripts increased to a peak at 6 h and declined again at 12 h. Twenty four hours after the ABA treatment, some genes were induced to a high level (such as *SlBBX16* and *17*) while some were down-regulated to a very low level (*SlBBX7, 12, 15*, and *25*). Upon 6-BA treatment (Figure 6D), the tomato BBX genes were more or less induced at the early stage, but the transcripts of most BBX genes decreased to a low level at 2 h, increased to a level as high as the early stage at 6 h, and down-regulated to an even lower level at 12 h. When treated with SA (Figure 6E), some genes were induced at the early stage but down-regulated later (such as *SlBBX2, 4, 5, 8*, etc.), while many of the BBX genes were expressed at a very high level 24 h after the treatment (such as *SlBBX3, 18, 19, 24, 25*, etc.). As response to MeJ (Figure 6F), most of the BBX genes were up-regulated at the early stage, but decreased at 6 h, and re-increased to a higher level at 24 h. Upon IAA treatment (Figure 6G), the changes of BBX gene expression were mild at the beginning, but almost all the genes were down-regulated at 12 h. After that time point, the expression level of some BBX genes increased to a peak (such as *SlBBX3, 14, 16, 17, 18, 19, 24*, and *28*), while for some other genes, it decreased to a bottom level, especially for *SlBBX7* and *SlBBX15*. Under ETH treatment (Figure 6H), most BBX members were inhibited at the initiation of the treatment, but they were induced more or less at the later stages. At 24 h, *SlBBX16, 17*, and *18* showed the highest expression level while *SlBBX7, 12*, and *15* showed the lowest transcript abundance.

### Differential expression of BBX genes under abiotic stresses

**Figure 7:**
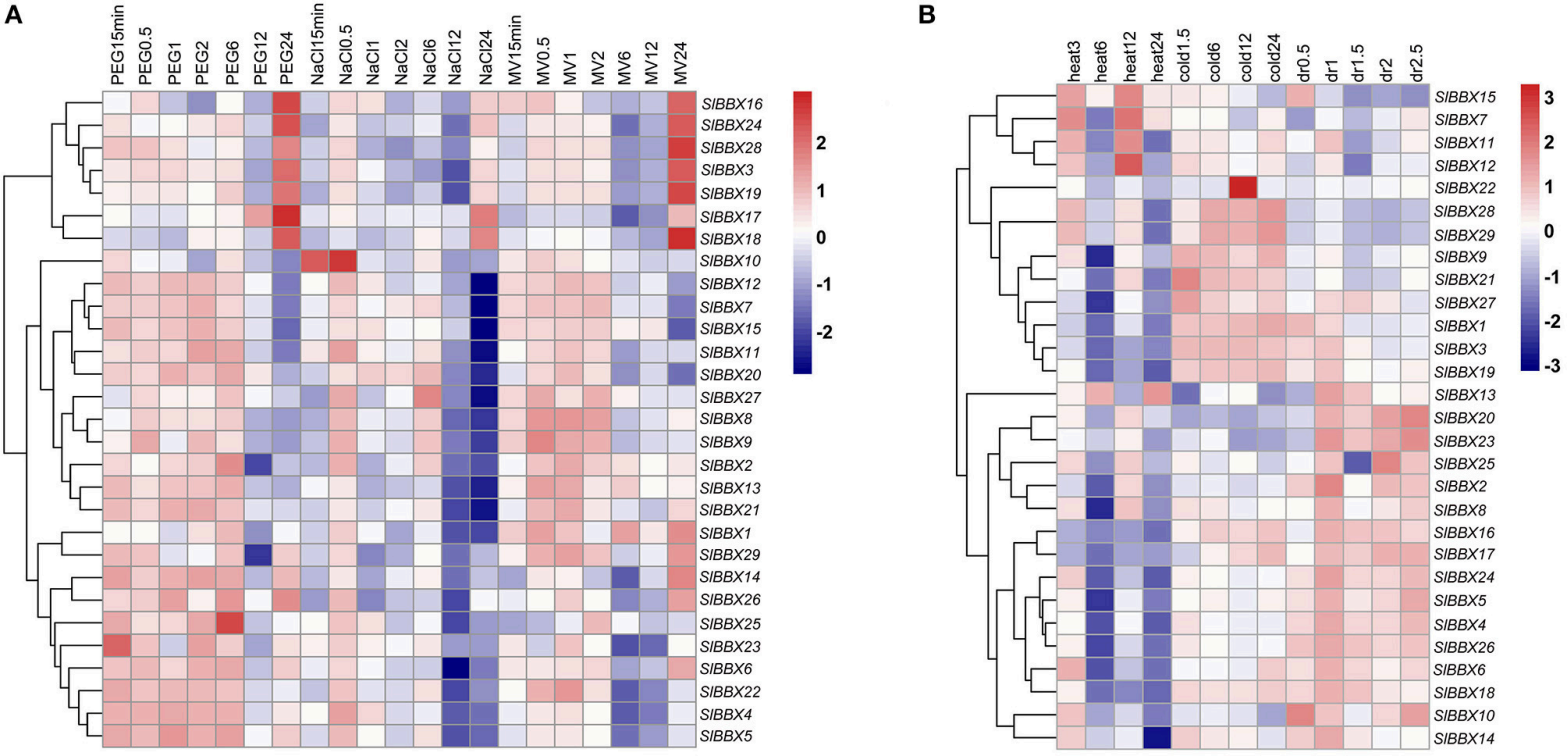
**Expression patterns of tomato BBX genes under different abiotic stresses**. The relative expression data were log2 transformed with R software, and a cluster dendrogram is shown on the left of each heat map. Blocks with blue colors indicate decreased and red ones indicate increased transcription levels. **(A)** shows the relative transcription level of BBX genes under different stress treatments in hydroponic culture condition. PEG, NaCl and MV represent seedlings treated with 10% polyethylene glycol-6000, 100 mM NaCl, and 5 μM Methylviologen, respectively. The numbers 0.5, 1, 2, 6, 12, and 24 indicate the time (hour) after treatments, and 15 min represents 15 min after treatment. Plants without stress (CK) at the same time are served as the control. **(B)** represents the gene expression patterns of the BBX genes treated with heat, cold, and dehydration stresses. For the heat stress, samples were collected at 3, 6, 12, and 24 h after 42°C treatment, and for the cold stress, samples were obtained at 1.5, 6, 12, and 24 h after 4°C treatment. Samples were taken at 0.5, 1, 1.5, 2, and 2.5 h after drought treatment (imitated with air drying the plants).

**Figure 8:**
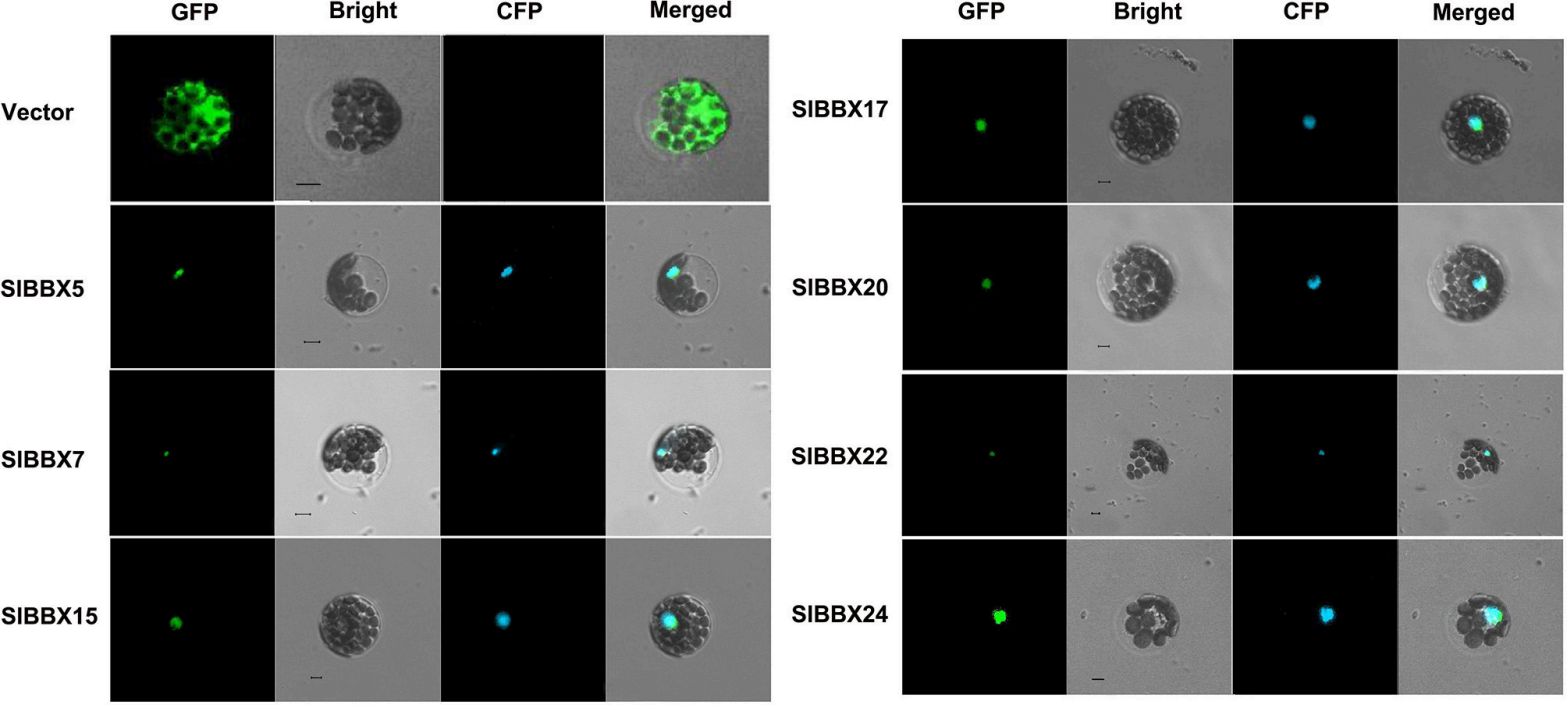
**Subcellular localization of seven GFP-fused tomato BBX proteins**. The plant expression vectors of green fluorescent protein (GFP) fused BBX proteins or control vector (pGWB451) were transformed into *Arabidopsis* protoplasts. The fluorescence was observed by confocal microscopy 24 h later. Nuclei were visualized by co-transformation of a cyan fluorescent protein (CFP) fused nucleus marker, Ghd7. Scale bar, 5 μm.

The expression profiles of BBX family genes under various abiotic stresses were also investigated, including polyethylene glycol 6000 (PEG), NaCl, methylviologen (MV), heat, cold, and drought treatment (Figure 7). Under PEG treatment (Figure 7A), most of the BBX genes showed elevated transcripts in the first 6 h, and most of them were down-regulated later and maintained at a low level. But the expression of several BBX genes reached their peak at 24 h, especially for *SlBBX16, 17, 18*, and *24*. Under NaCl stress (Figure 7A), the changes for most BBX genes were mild, except for *SlBBX10*, which was obviously up-regulated within half an hour. At the later stages of salt stress, most BBX genes were severely suppressed by NaCl stress (Figure 8A), and only a few of them showed an increased expression at 24 h (such as *SlBBX17* and *18*). Most of the BBX genes were more or less induced by the MV treatment within the first 2 h, but they were down-regulated at 6 and 12 h. At 24 h, *SlBBX3, 16, 18, 19, 24*, and *28* showed a high expression level upon MV stress (Figure 7A). When the plants were submitted to heat stress (Figure 7B), most BBX genes were down-regulated at 6 and 24 h, and changed only slightly at the other two time points. Four BBX genes (*SlBBX7, 11, 12, 15*) were expressed highly at 3 and 12 h, and they were down-regulated at 6 and 24 h. In addition, there was another gene (*SlBBX13*) which appeared different, and it showed a high expression level at 6 and 24 h but had a reduced transcription level at 3 and 12 h after heat treatment. When the plants were challenged with cold stress (Figure 7B), only eight genes (*SlBBX1, 3, 9, 19, 21, 27, 28*, and *29*) showed an increasing pattern in expression. Upon to drought stress treatment (Figure 7B), most BBX genes showed a relatively higher expression level, such as *SlBBX4, 5, 6, 16, 17, 20, 23, 24*, and *26*.

### Subcellular localization of tomato BBX proteins

The subcellular localization of tomato BBX proteins were firstly predicted using WoLF PSORT (Table 1). Twenty-two of them had a high probability to be located in nucleus. However, SlBBX5, 6, 23, and 28 were presumably located in chloroplast, and SlBBX16, 18, and 19 in cytosol. To verify this, seven genes (*SlBBX5, 7, 15, 17, 20, 22*, and *24*) which were strongly responsive to hormones, abiotic stresses or specifically expressed in some organs, were selected for a transient expression assay using *Arabidopsis* mesophyll protoplasts. When the GFP-fused BBX proteins were co-expressed with the CFP-fused nucleus marker (Ghd7), the GFP signal was visualized only in the nucleus, while the control GFP protein (GFP gene driven by the 35S promoter) was observed throughout the protoplast (Figure 8). These results were in consistent with the prediction results, except for *SlBBX5* (Table 1) which was predicted to be preferentially located in chloroplast, than in nucleus.

## Discussion

### Evolution of the tomato BBX gene family

The present study characterized the structure, phylogenetic relationship, subcellular localization, duplication events and expression profiles of the whole BBX gene family in tomato. BBXs can be divided into five subfamilies according to the sequence similarity (Gangappa and Botto, 2014). The number of BBX genes is relatively consistent among different plant species (Table S5). As reported, *Arabidopsis* and rice possess 32 and 30 BBX genes, respectively (Khanna et al., 2009; Huang et al., 2012). We found cultivated and wild tomato species (*S. pennellii*) have the same number of BBX genes, with 29 members each. We also retrieved the BBX genes from pepper and potato genomes. Pepper has a much larger genome than tomato, however, the number of BBX genes could be even less than that of tomato. The only exception is potato, which had 49 BBX genes according to our analysis, this could be accounted for that cultivated potato is a highly heterozygous tetraploid species. The genome composition of potato is much more complex than that of tomato, though homozygous doubled-monoploid potato has been used in the genome decoding (Potato Genome Sequencing Consortium, 2011).

Although the difference in number is small, the composition of different classes of BBX genes is different among species (Table S5). In *Arabidopsis*, the numbers of BBX members with B-BOX1 only, two tandem B-BOXes, BOX1 plus CCT, two tandem B-BOXes plus the CCT domain are 7, 8, 4, and 13, respectively. The corresponding numbers were 6, 10, 5, and 8 in tomato. The 32 BBX members of *Arabidopsis* can be clearly divided into five classes based on the combination of different conserved domains (Khanna et al., 2009). AtBBX1 to AtBBX6 belongs to class I, and AtBBX7 to AtBBX13 belongs to class II. All the members in these two classes contain two B-BOXes plus the CCT domain. AtBBX14 to AtBBX17 are in class III, which have the BOX1 and the CCT domains. AtBBX18 to AtBBX25 represent class IV, which carries both B-BOX1 and B-BOX2. The rest members (AtBBX26 to AtBBX32) possess the B-BOX1 domain only. The classification of tomato BBX members based on conserved domain was relatively difficult. As shown in Figure 2, seven BBX members were classified into class III, however, among these BBX proteins, SlBBX9, SlBBX11 and SlBBX12 possessed all the three domains, SlBBX8 and SlBBX10 contained B-BOX1 and the CCT, SlBBX7 carried with the two tandem B-BOXes, while SlBBX27 with B-BOX1 only. None of the corresponding genes were involved in tandem or segmented duplication events. Similar classification results are also obtained in rice, and it was speculated that the B-BOX2 domain in some of the BBX proteins is deleted in the evolution process (Griffiths et al., 2003). In addition, the clustering result based on full-length amino acid sequences of the 29 BBX members was similar to that based on B-BOX1 (Figures 2A,B), in which SlBBX7, 8, 9, 10, 11, 12, and 27 were also clustered together. When we checked the detail of sequence alignment (Figure S2), it was found that the B-BOX1 domain was highly conserved among the seven members. This could be explained by a previous review, which pointed out that a deletion event could happen on the B-BOX2 domain of an early BBX member belonging to class II, thus rises to a BBX protein in class III with a single B-BOX (Crocco and Botto, 2013). Therefore, it was difficult to group the family members contain two B-BOXes and one CCT domain into one subfamily, which was similar to the classification in the previous study in rice (Huang et al., 2012).

BBX proteins in *Arabidopsis* are key factors in regulating plant growth and developmental processes, including seedling photomorphogenesis, photoperiodic regulation of flowering, shade avoidance, and response to biotic and abiotic stresses (Gangappa and Botto, 2014). Specific roles of the tomato BBX genes in plant growth and stress response remain to be elucidated.

### Organ-specific and stress induced expression of *SlBBX* genes

BBX genes are involved in seedling photomorphogenesis including hypocotyl growth, chlorophyll accumulation, and cotyledon unfolding processes, flowering, and abiotic or biotic stresses in *Arabidopsis* or rice (Datta et al., 2007, 2008; Gangappa and Botto, 2014). However, expression profiles of tomato BBX family have not been demonstrated before. Here, the transcript profiles of 29 *SlBBXs* were investigated in nine tomato organs. Suppressing the expression of *BBX24* from *Chrysanthemum via* transgenic approach results in earlier flowing and decreased tolerance to drought and freezing tolerance (Yang et al., 2014). *SlBBX24* may play similar roles in tomato, as it was the only gene constitutively and highly expressed in all organs, and it was also induced by drought stress treatments (PEG-6000 or dehydration). In *Arabidopsis*, AtBBX18 (known as DBB1a) regulates a series of genes playing fundamental role in floral development, a defect in this protein causes abnormal floral development (Wang et al., 2009). Besides *SlBBX18*, three other tomato BBX genes (*SlBBX6, 17*, and *19*) were also expressed at a relatively higher level in flower. One BBX gene, *SlBBX20*, was expressed at a high level in tomato fruits at different stages, suggesting its potential role in fruit development.

Various abiotic stresses, including drought, salinity, oxidative stress and extreme temperatures can induce a range of stress response mechanisms, and then activate related genes needed for stress tolerance. In this study, 28 tomato BBX genes possessed at least one of the stress response *cis*-elements (HSE, TC-rich, MBS, and ARE) in their promoter regions, implying their potential roles in stress response. Actually, four genes (*SlBBX7, 11, 12*, and *15*) were induced upon heat stress, while seven other members (*SlBBX1, 3, 9, 19, 21, 28*, and *29*) were activated by cold stress (Figure 7B). All these genes contained HSE or TC-rich elements in their promoters. There were ten *SlBBXs* (*SlBBX2, 4, 5, 8, 13, 14, 20, 23*, and *25*) possessing the MBS or TC-rich *cis*-elements in their promoters, all of them exhibited higher expression levels under PEG and drought treatments, except for *SlBBX6*. Gong et al. (2010) has found that *SlBBX20* is up-regulated in M82 when the plants were challenged with drought stress. Similar expression patterns of *SlBBX20* were identified in the two tomato species tested in this study, indicating its important roles in drought responsiveness.

### Hormone responsive expression of *BBXs* in tomato

Plant hormones are originally characterized as regulators in growing and developmental processes, simultaneously, the evidence for the role of BBX proteins in hormonal signaling pathways is scarce (Hirano et al., 2007; Bari and Jones, 2009; Pieterse et al., 2012). In a previous study, 11 BBX genes in rice show differential expression levels when exposed to auxin, GA and cytokinin, and most of them harbor hormone-responsive *cis*-elements in their promoters. In our study, five hormone-responsive *cis*-acting elements were identified in tomato BBX gene promoters. The expression of *SlBBX4, 5, 21*, and *22* was induced by ETH, and all of them contained the ethylene-responsive *cis*-element (ERE) in their promoters. Similarly, *SlBBX7* and *SlBBX12* were up-regulated by ABA, heat, PEG, and MV treatments and they harbored abscisic acid responsive element in their promoters. It was suggested that these BBX genes may be involved in hormone signaling as transcriptional regulators to modulate plant tolerance response to abiotic stresses.

### The different expression patterns of BBX genes between *S. pennellii* and M82

Previously, several BBX genes (*SlBBX3, 11, 14, 20*, and *24*) have been identified to be differentially expressed between a drought-tolerant introgression line (IL2-5) of *S. pennellii* LA0716 and its recurrent parent M82, indicating their potential roles in the drought stress response of tomato (Gong et al., 2010). Furthermore, according to the chromosomal location of *SlBBXs* in tomato, three BBX genes (*SlBBX1, 2, 3*) were located in the introgressed region of IL2-5. *S. pennellii* LA0716 is well-known as a stress-tolerant wild tomato accession (Bolger et al., 2014), while the cultivated tomato M82 is stress-sensitive. In order to elucidate their biological functions, the transcript patterns were compared between M82 and LA0716 under drought treatment. Obviously, the expression patterns of many BBX genes were different between these two species, such as *SlBBX1, 2, 4, 5, 10, 18, 26*, and *28*, which were induced upon drought treatment in M82 whereas they were down-regulated after 2.5 h drought treatment in LA0716. This suggested these genes might play an essential role in the different performance of the two tomato species under stress condition.

In this study, a comprehensive analysis of the 29 *SlBBX* genes in tomato genome was performed, including the gene structure, subcellular localization predication, phylogenetic and duplication analysis, and *cis*-regulatory elements prediction. The transcription of some *SlBBXs* can be induced by hormones, abiotic stresses, or differentially expressed in different organs, which indicated these BBX members may have various functions in physiological activities. In addition, the subcellular location of seven BBX members were confirmed by the transient expression assay, it was suggested that they might act as transcription factors to regulate the transcription of other genes in nucleus. All of these data will lay a solid foundation for functional characterization of BBX genes in tomato, and further study on several BBX genes is undergoing to understand their biological functions.

## Author contributions

ZC, JL, YL (Y Lu), BO participated in the design of the study; ZC analyzed the data, and wrote the manuscript; XW, HY assisted with bioinformatic analysis and sequences alignment; YL (Y Li) participated in subcellular localization and data analysis; BO initiated and supervised the study and wrote the manuscript. All authors read and approved the final version.

### Conflict of interest statement

The authors declare that the research was conducted in the absence of any commercial or financial relationships that could be construed as a potential conflict of interest.

## Supplementary material

Table S1 **Primers used for qPCR analysis**.

Table S2 **The amounts of the ***cis***-elements that were identified in more than 10 ***BBX*** genes in tomato**.

Table S3 **Hydroponic solution for tomato culture**. “MW” means the molecular weight. “μM,” “mM,” and “M” represent the concentration units were μM/L, mM/L, and M/L, respectively.

Table S4 **The differences about the ***cis***-elements in the promoter sequences of BBXs between ***S. pennellii*** and M82**. Yellow (blue) color means the *cis*-elements appear in the BBX promoters of *S. pennellii* (M82), but not in M82 (*S. pennellii*).

Table S5 **BBX gene family in six species**. “BOX1” indicates the BBX members only contain BOX1 domain; “BOX1+BOX2” represents the BBX members contain BOX1 domain plus BOX2 domain; “BOX1+CCT” indicates the BBX members contain BOX1 domain and CCT motif; and “BOX1+BOX2+CCT” means the BBX members possess two BOX domains plus CCT domain.

Figure S1 **Neighbor-joining tree of BBX proteins from tomato, ***Arabidopsis*** and rice**. The BBXs with “At,” “Os,” and “Sl” represent from *Arabidopsis*, rice, and tomato, respectively. The triangle marks in different colors (red for *Arabidopsis*, blue for rice, and black for tomato) mean the BBX proteins contain two B-BOXes and one CCT domain.

Figure S2 **Alignment of the conserved domains of tomato BBX proteins**. Multiple sequence alignments of the B-BOX1 **(A)**, B-BOX2 **(B)**, and CCT **(C)** domain are shown. Completely conserved residues in a domain are indicated by black boxes, while residues conserved in the majority of sequences are indicated by gray boxes.

Figure S3 **Expression patterns of tomato BBX genes upon drought stress in cultivated tomato M82 and wild species ***S. pennellii*** (LA0716)**. Hierarchical clustering of the relative transcript abundance profiles (log2 scale) of the BBX members was performed with R software. Blocks with blue colors indicate decreased and red ones indicate increased transcription levels. The numbers 1 and 2.5 indicate the time (h) after treatments. Plants without stress (CK) at the same time were served as the control.

## Abbreviations

ABA: Abscisic acid
BR: Brassinolide
CFP: Cyan fluorescent protein
ETH: Ethephon
GA: Gibberellic acid
GFP: Green fluorescent protein
IAA: Auxin
IL: Introgression line
MeJ: Methyl jasmonate
MV: Methylviologen
PEG: Polyethylene glycol-6000
qPCR: Real-time quantitative PCR
SA: Salicylic acid
6-BA: 6-Benzylaminopurine
CO: Constans
FT: Flowering locus T.
