# Supplementary figures and images for: Genomic Organization, Phylogenetic and Expression Analysis of the B-BOX Gene Family in Tomato
Source: Front Plant Sci. 2016 Oct 19;7:1552. doi: 10.3389/fpls.2016.01552 (PMC5069294; doi:10.3389/fpls.2016.01552)

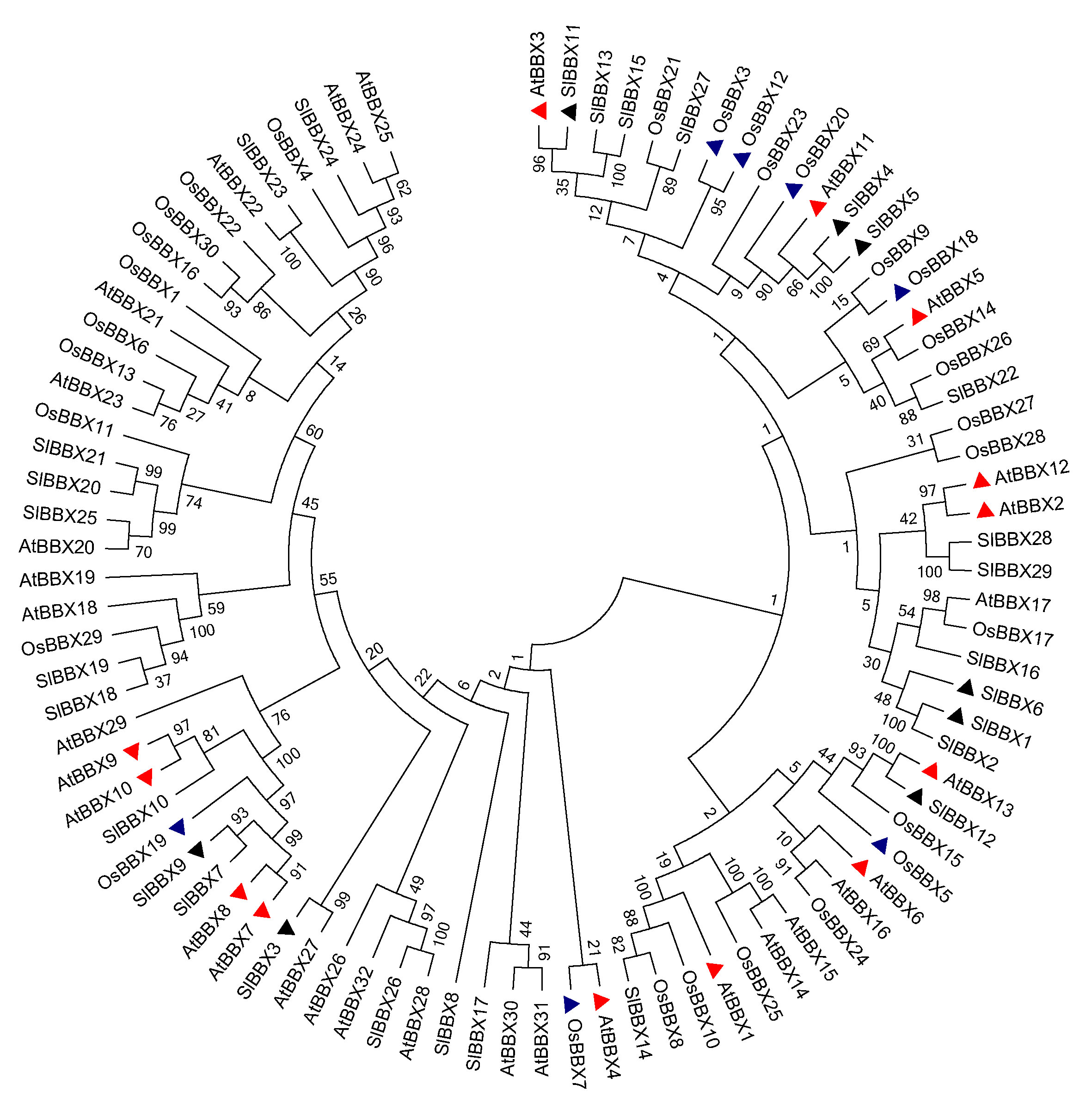

Supplement: Figure S1 — Neighbor-joining tree of BBX proteins from tomato, Arabidopsis and rice. The BBXs with “At,” “Os,” and “Sl” represent from Arabidopsis, rice, and tomato, respectively. The triangle marks in different colors (red for Arabidopsis, blue for rice, and black for tomato) mean the BBX proteins contain two B-BOXes and one CCT domain. [file Image1.JPEG]

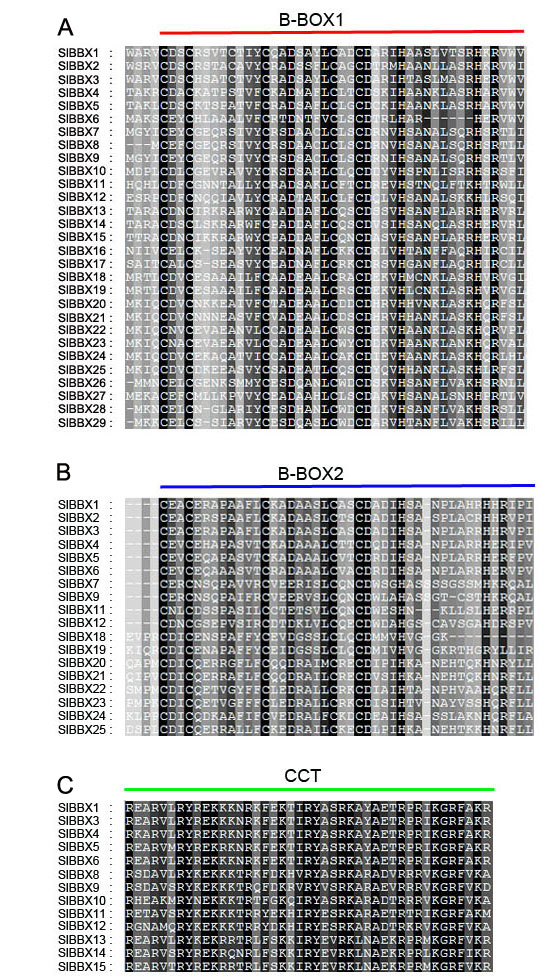

Supplement: Figure S2 — Alignment of the conserved domains of tomato BBX proteins. Multiple sequence alignments of the B-BOX1 (A), B-BOX2 (B), and CCT (C) domain are shown. Completely conserved residues in a domain are indicated by black boxes, while residues conserved in the majority of sequences are indicated by gray boxes. [file Image2.JPEG]

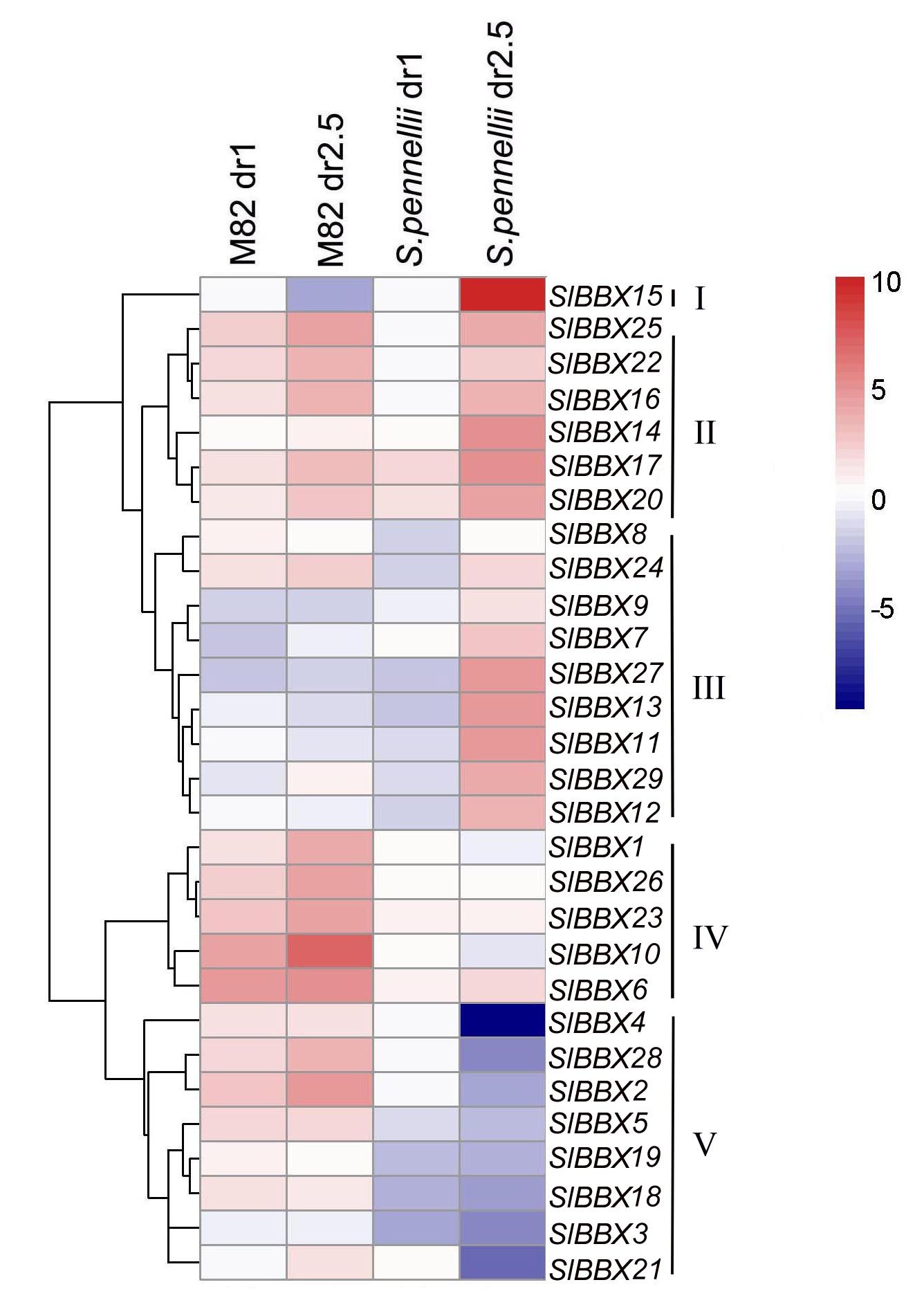

Supplement: Figure S3 — Expression patterns of tomato BBX genes upon drought stress in cultivated tomato M82 and wild species S. pennellii (LA0716). Hierarchical clustering of the relative transcript abundance profiles (log2 scale) of the BBX members was performed with R software. Blocks with blue colors indicate decreased and red ones indicate increased transcription levels. The numbers 1 and 2.5 indicate the time (h) after treatments. Plants without stress (CK) at the same time were served as the control. [file Image3.JPEG]
